# Molecular profile and response to energy deficit of leptin-receptor neurons in the lateral hypothalamus

**DOI:** 10.1038/s41598-022-16492-w

**Published:** 2022-08-04

**Authors:** N. Kakava-Georgiadou, V. Drkelic, K. M. Garner, M. C. M. Luijendijk, O. Basak, R. A. H. Adan

**Affiliations:** 1 Department of Translational Neuroscience, Division of Neuroscience, UMC Brain Center, University Medical Center Utrecht, Utrecht University, Utrecht, The Netherlands; 2 Institute of Neuroscience and Physiology, The Sahlgrenska Academy at the University of Gothenburg, Gothenburg, Sweden

**Keywords:** Molecular biology, Neuroscience

## Abstract

Leptin exerts its effects on energy balance by inhibiting food intake and increasing energy expenditure via leptin receptors in the hypothalamus. While LepR neurons in the arcuate nucleus of the hypothalamus, the primary target of leptin, have been extensively studied, LepR neurons in other hypothalamic nuclei remain understudied. LepR neurons in the lateral hypothalamus contribute to leptin's effects on food intake and reward, but due to the low abundance of this population it has been difficult to study their molecular profile and responses to energy deficit. We here explore the transcriptome of LepR neurons in the LH and their response to energy deficit. Male LepR-Cre mice were injected in the LH with an AAV carrying Cre-dependent L10:GFP. Few weeks later the hypothalami from fed and food-restricted (24-h) mice were dissected and the TRAP protocol was performed, for the isolation of translating mRNAs from LepR cells in the LH, followed by RNA sequencing. After mapping and normalization, differential expression analysis was performed with DESeq2. We confirm that the isolated mRNA is enriched in LepR transcripts and other known neuropeptide markers of LepR^LH^ neurons, of which we investigate the localization patterns in the LH. We identified novel markers of LepR^LH^ neurons with association to energy balance and metabolic disease, such as *Acvr1c*, *Npy1r, Itgb1*, and genes that are differentially regulated by food deprivation, such as *Fam46a* and *Rrad*. Our dataset provides a reliable and extensive resource of the molecular makeup of LH LepR neurons and their response to food deprivation.

## Introduction

Leptin is a hormone secreted by white adipose tissue proportional to the amount of stored fat. It regulates several aspects of energy balance such as food intake and energy expenditure^1–5^. Fasting decreases and feeding increases plasma leptin levels^6^. Despite elevated plasma leptin levels in people with obesity^7^, leptin fails to reduce energy intake and increase energy expenditure, which is referred to as leptin resistance^8^. Leptin exerts its effects on energy homeostasis mainly via cells expressing leptin receptor (LepR) in the hypothalamus^9^. When developing obesity, these cells fail to respond to leptin and therefore they are potential targets with therapeutic potential in eating disorders. The most prominent population of LepR-expressing cells reside in the arcuate nucleus (Arc) of the hypothalamus where leptin stimulates pro-opiomelanocortin (POMC) neurons and inhibits Agouti-related peptide (AgRP) neurons, the latter responding highly to energy deficit^10–12^. LepR cells in other brain sites such as in the lateral hypothalamus (LH) are less studied.

The lateral hypothalamus (LH) is a hub of feeding behavior and of the reward system, containing distinct neuropeptide populations as well as passing fibers^13,14^. The LH plays a critical role in connecting energy needs with motivation to eat and is therefore of interest to explore the relation between overconsumption and development of obesity. LepR is expressed in Galanin- and Neurotensin-expressing populations of GABAergic neurons in the LH, which regulate food intake, body weight and connect the status of energy balance to the dopaminergic reward system^15,16^. We here aimed to unravel the molecular profile of the populations of LepR^LH^ neurons as well as their transcriptomic response to food restriction, using TRAP-Seq^17^.

We used LepR-cre mice to study the transcriptome of neurons that express the long isoform (LepRb) of the leptin receptor. These cells will, besides leptin, respond to a variety of hormones and neuropeptides. TRAP is an RNA polysome immunopurification technique which facilitates the isolation of mRNA bound to GFP-tagged ribosomes, from cells that artificially express GFP fused to the ribosomal subunit L10^18^. TRAP-Seq of the total population of hypothalamic LepR cells already revealed a molecular signature^19^. However, by looking at the whole hypothalamic population of LepR cells as bulk, the large population of LepR neurons in the arcuate nucleus dominates the results. To target LepR neurons in the LH, we employ viral TRAP to isolate mRNA exclusively from LepR^LH^ neurons. Therefore, we provide an extensive and reliable resource of LepR^LH^-enriched genes as well as genes differentially regulated by energy deficit, associated with energy balance and metabolic disease.

## Results and discussion

In order to isolate mRNA from LepR^LH^ cells, an AAV carrying a Cre-dependent cDNA for L10:GFP was injected into the LH of LepR-Cre adult male fed and fasted mice and 4 to 5 weeks later hypothalami were dissected and TRAP was performed, a timepoint that allows sufficient viral expression and detection of L10a:GFP in polysome fractions^17^. cDNA libraries were prepared with the CelSeq2 protocol optimized for bulk RNA and the libraries were sequenced with next generation sequencing (NGS). Analysis of the RNA-sequencing reads revealed enrichment of genes in the LepR^LH^ population against the total hypothalamus as well as genes differentially regulated by food deprivation.

### Validation of selective mRNA enrichment from LepR^LH^ neurons

**Figure 1:**
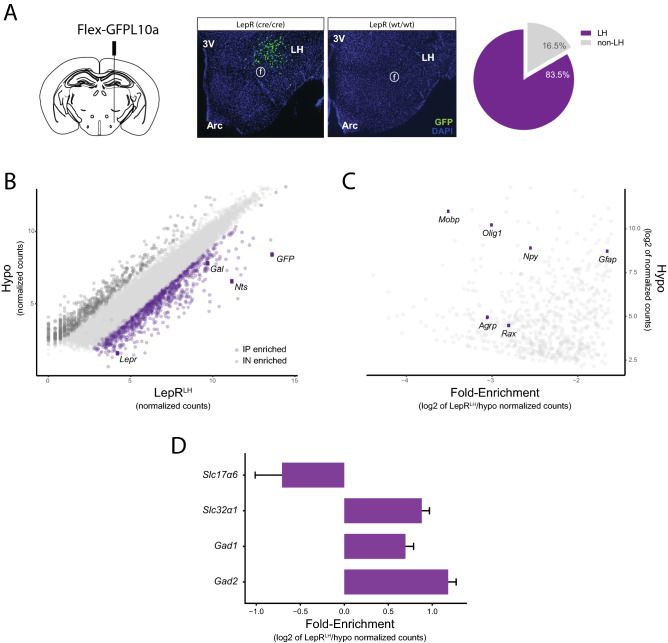
Targeting and RNA enrichment from LepR^LH^ cells (**A**) LepR-Cre mice and wild type mice were injected in the LH with an AAV carrying Flex-L10:GFP (graphic of coronal section does not represent exact coordinates). In wild-type littermates (wt/wt) the GFP expression was very low (right image) compared to LepR-cre mice. In homozygous Cre (cre/cre) mice, 83.5% of the cells with immunohistochemically-detected GFP were in the LH (mean, n = 12 hypothalami). (**B**) Scatterplot of RNAseq results showing normalized counts from LepR^LH^ fraction (IP) in x axis plotted against normalized counts from Hypothalamus fraction (IN) in y axis. Violet-colored dots represent genes that are enriched in IP (with log2FoldChange > 1.5 and adjusted *p* < 0.01) and dark grey-colored dots represent genes that are enriched in IN (with log2FoldChange < − 1.5 and adjusted *p* < 0.01). GFP, LepR and known marker genes of LepR^LH^ cells are represented as dots colored in dark violet. (**C**) Scatterplot of RNAseq results showing genes de-enriched in IP fraction (enriched in IN fraction) as log2 of normalized counts from IN fraction (x-axis) plotted against Fold-enrichment (log2 of IP/IN normalized counts) (y-axis). Markers known to not be present in LepR^LH^cells (*Agrp*, *Npy*) and non-neuronal markers are represented as dark violet dots. (**D**) Barplot showing Fold-enrichment (log2 of IP/IN normalized counts) of GABAergic markers *Slc32a1*, *Gad1* and *Gad2* and glutamatergic marker *Slc17a6* (mean ± SEM). IP: immunoprecipitated samples, IN: input samples.

A series of pilot experiments were performed in which different serotypes and virus titers of rAAV-flex-L10:GFP were stereotaxically injected into mouse brains with several coordinates targeting the LH, in order to achieve sufficient GFP expression targeted in the LH. GFP expression was observed in the LH of LepR-Cre (cre + /cre +) mice and it was minimal in wild-type (wt/wt) littermates (Fig. 1a—left). The TRAP experiment was performed in 7 batches of male mice, both from the fed and fasted condition. In each batch few mice were sacrificed before performing TRAP in order to assess GFP expression (see Methods for details). Data from a total of 12 mice, showed 1256.7 ± 215.7 GFP-expressing cells per mouse, of which 83.5% ± 3.8 were localized in the LH (Fig. 1a—right, see Methods). Viral spread upon injection in the LH caused a small proportion of labeled cells outside the LH.

Immunoprecipitated RNA from LepR^LH^ cells (**LepR**^**LH**^) as well as RNA from the whole hypothalamus (**hypo**) was sequenced. Following normalization of sample by Deseq2 (Median of ratios method) to an average of ~ 3,785,000 reads per sample and exclusion of lowly expressed genes (a cutoff of 5 reads per sample), we obtained a final dataset containing 13,093 of the 17,988 genes detected in the dataset. Differential expression analysis between LepR^LH^ and whole hypothalamus (hypo) samples identified 768 genes with significantly different expression levels (with adjusted *p* < 0.01) (sup. Table 1). 343 genes (including GFP) were enriched in LepR^LH^ over hypo (log2FoldChange > 1.5) and 425 genes were enriched in hypo over LepR^LH^ (log2FoldChange < − 1.5) (Fig. 1b). As expected, *GFP* and *LepR* were both enriched in LepR^LH^ over hypo (38.3-fold and 6.4-fold respectively, *p* < 0.01, Fig. 1b) with *GFP* being the most significantly enriched gene of the whole dataset. The well-established markers of LepR^LH^ neurons, *Gal* and *Nts*, were also enriched in LepR^LH^ over hypo (3.8-fold and 24.2-fold respectively, *p* < 0.01, Fig. 1b). We detected a significant enrichment of lateral hypothalamic genes by comparing the 342 genes (excluding GFP) enriched in LepR^LH^ with upregulated genes across brain regions provided by the Allen Brain Atlas using the Enrichr platform (sup. Table 2), confirming effective targeting of the LH. Thus, our dataset identifies the molecular makeup of the LepR^LH^ neurons.

**Table 1:** Probes used in FISH.

Gene	Forward primer (5'- > 3')	Reverse primer (5'- > 3')
*Cartpt*	GCGCTATGTTGCAGATCGAA	ACATGCTTCAATTTGTGTGGCT
*Crh*	TGCGTGCTTTCTGAAGAGGG	TTTTGGCCAAGCGCAACATT
*Gal*	ATCCAGCCCGCCACTCTTCA	ACAGCTTCAAAGCAGAGAACAGA
*Nts*	AGAAGAAGATGTGAGAGCCCTG	TGCTTTGGGTTAATAACGCTCC
*Tac1*	ATGAAAATCCTCGTGGCCGT	TCATACAATGACTGAAGACCAGAGA

Next, we asked whether specific cell types are enriched in LepR^LH^ neurons compared to the whole hypothalamus. Non-neuronal markers, such as *Gfap* for astrocytes, *Mobp* and *Olig1* for oligodendrocytes, *Cx3cr1* for microglia, and *Rax* for tanycytes, as well as markers of distinct hypothalamic populations, such as *Agrp* and *Npy* (Fig. 1c), and LH-specific *Pmch* and *Hcrt* (not shown) were less abundant in IP (IP = immunoprecipetated fraction enriched in LepR^LH^) than IN (IN = input RNA from all cells of LH) samples. RNA enriched from LepR LH cells showed enrichment of GABAergic markers *Slc32a1*, *Gad1* and *Gad2*, and low levels of glutamatergic marker *Slc17a6*, showing that we enriched for GABAergic LepR^LH^ neurons (Fig. 1d). These results also confirm the literature that has extensively established LepR^LH^ neurons as GABAergic^15^.

### Enrichment and localization of established markers of LepR^LH^ neurons

Besides their established GABAergic nature, literature has shown the expression of a handful of neuropeptides and receptors in LepR^LH^ neurons. LepR neurons in the LH express Neurotensin (*Nts*), Galanin (*Gal*), CART peptide (*Cartpt*), Tachykinin 1 (*Tac1*) as well as Corticosterone-releasing hormone (*Crh*)^15,19,20^. In the LH, LepR neurons have been reported to express melanocortin receptors *Mc3r* and *Mc4r* as well the receptor for calcitonin *Calcr*^21–23^. We confirmed the enrichment of all of these genes in the IP fraction (immunoprecipetated fraction enriched in LepR^LH^) over IN (input RNA from all cells of LH) in our dataset (Figs. 1b and 3). Moreover, we show that neuropeptides Calca, Cbln2, Pomc and Vgf were also enriched in LepR^LH^ neurons compared to the whole LH (Fig. 3).

**Figure 2:**
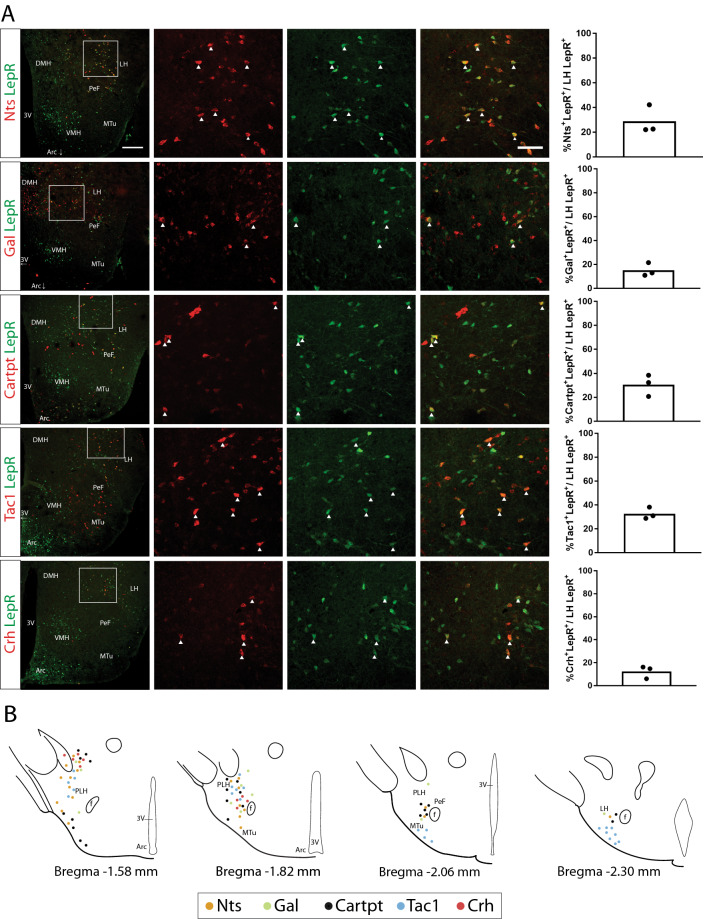
Expression of neuropeptides in LepR^LH^ cells and co-localization patterns in the LH. (**A**) Fixed coronal sections from LepR-Cre x L-tdTomato mice underwent fluorescent in situ hybridization (FISH) with anti-sense RNA probes for *Nts*, *Gal*, *Cartpt*, *Tac1* and *Crh* ("*Neuropeptide*"—red channel) followed by immunohistochemistry (IHC) for tdTomato ("LepR"—green channel) and imaging. The percentage of *Neuropeptide*^+^LepR^+^ cells out of the total LepR^+^ in the LH was quantified, as represented in the barplots (mean, n = 3 hypothalami). (**B**) Localization of *Neuropeptide*^+^LepR^+^ cells was observed under the microscope and drawn on representative graphic images of coronal sections from the mouse Brain Atlas^58^, which were adapted for this figure. Arc: Arcuate nucleus of the Hypothalamus, 3 V: 3rd Ventricle, DMH: Dorsomedial Hypothalamus, VMH: Ventromedial Hypothalamus, LH: Lateral Hypothalamus, PeF: Perifornical area, f: Fornix, MTu: Medial Tuberal nucleus.

We next determined the co-localization pattern of the five neuropeptides with LepR in the LH. To this end, we performed fluorescent in situ hybridization (FISH) for *Nts*, *Gal*, *Cartpt*, *Crh* and *Tac1* on tissue from LepR-tdTomato mice stained for tdTomato. We focused on the LH (including the Pernifornical area (PeF), Zona Incerta (ZI), Tuberal nucleus (Tu)) and explored co-localization of markers with tdTomato (LepR). *Cartpt, Nts* and *Tac1* were expressed in almost one third of LepR cells in the LH, while Gal and Crh were expressed in 15% and 12% of the cells respectively (Fig. 2a). Literature shows that more than half (60%) of LepR^LH^ neurons express the anorexigenic peptide Neurotensin (*Nts*), while 20–44% of pSTAT3 positive cells in the LH express Galanin^15,20^. The difference observed between the two sets of data can be attributed to different detection methods used—for example, not all of LepR cells could be engaged in pSTAT3 signaling at the time of the experiment, while tdTomato expression is stable—or to the fact that we included the ZI and Tu in our measurements.

Co-localization of each marker with LepR^LH^ cells was observed in the dorsal part of the LH/Zona Incerta (ZI), Pernifornical area (PeF) and ventral part of the LH/tuberal nucleus (Fig. 2b). Based on literature, Neurotensin is almost fully co-expressed (95%) with Galanin, and CART peptide is expressed in half (56%) of Galanin positive neurons in the LH^20^. In line with this, we observed that *Gal* co-localizes its expression with LepR in the same subregions as *Nts* as well as *Cartpt*. In the case of *Crh*, co-localization is observed in the rostral part of the LH and PeF. Therefore, it is highly probable that all three neuropeptides could have overlapping expression in the same cell, while we did not observe a distinct pattern of *Gal*, *Nts* and *Crh* expression in LepR^LH^ neurons.

Even though *Tac1* and *Cartpt* co-localized with LepR in the rostral part of the LH/ZI and PeF, we also observed distinct patterns of expression for them. We observed *Cartpt*/LepR cells in the ventral LH/MTu rostrally (Bregma − 1.58), while there were *Tac1*/LepR cells also localized in the ventral LH/MTu caudally (Bregmas − 2.06 and − 2.30). These findings suggest that in contrast to the Arc, where LepR is mostly expressed in distinct populations of neurons and has directly opposing functions on Agrp and POMC neurons, LepR in the LH is found in neurons with diverse profiles, with overlapping as well as distinct expression of neuropeptides. Therefore, leptin may act on a variety of distinct neurons with partially overlapping genetic identity.

### Novel markers of LepR^LH^ neurons

**Figure 3:**
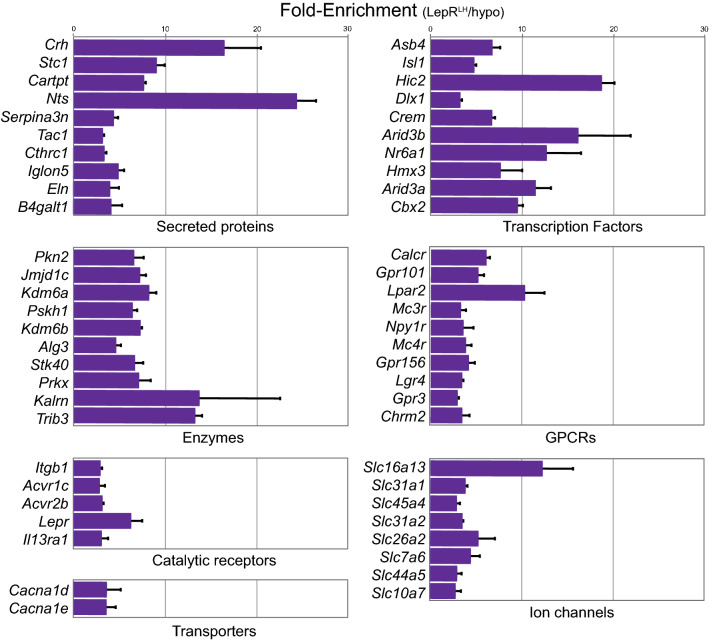
Genes significantly enriched in IP over IN fraction categorized by molecular function. Barplots represent RNAseq results of the Fold-enrichment (IP/IN of normalized counts) in x-axis of the top 10 most significantly enriched genes between the IP and IN fractions (mean ± SEM) per type of molecular function (sorted by adjusted *p* value).

We next focused on novel markers of LepR^LH^ neurons. Using the Uniprot database, we assigned the TRAP-enriched genes to gene ontology and molecular function terms (sup. Table 3). Where possible, LepR^LH^-enriched genes were split into main sub-categories based on their molecular function: secreted proteins, enzymes, catalytic receptors, GPCRs, ion channels, transporters and transcription factors (see Column "Type" in sup. Table 3 and Methods for databases used for annotation). We continued with the top 10 significant genes of each category (Fig. 3). The genes were analyzed for their expression in the hypothalamus and particularly the LH, based on ISH data of Allen Brain Atlas (referred to as **ABA** from now on) and mouse **GTEx** data from the protein atlas. Moreover, in order to assess relevance, we performed literature search (PubMed) using as search terms the gene name combined with either hypothalamus, diabetes or obesity as keywords.

### LepR^LH^ -enriched markers with selective expression in the LH and other hypothalamic nuclei

We identified genes with selective expression in the LH and other hypothalamic nuclei where LepR is expressed, based on Allen Brain Atlas ISH data (ABA).

#### Catalytic receptors

*Itgb1*, which codes for β1 integrin associates with integrins α1 and α2 to form integrin complexes which function as collagen receptors. It shows prominent expression in the LH, tubelar nucleus (Tu), PVN and weakly in Arc (ABA), but its function in the hypothalamus remains unexplored. *Acvr1c* (also known as Alk-7) is expressed in the Arc, VMH and weakly in the LH (ABA) and is downregulated in adipose tissue in obese subjects^24^, while variations in its DNA sequence are associated with protection against diabetes type 2^25^. Besides being enriched in Agrp neurons in the Arc^12^, nothing is known about the function of *Acvr1c* in the hypothalamus.

#### Secreted proteins

The secreted protein *Stc1* codes for Stanniocalcin 1, involved in lipid synthesis in brown adipose tissue^26^ and its mRNA is expressed very weakly in the LH and DMH in the hypothalamus (ABA). Another secreted protein, *Cthrc1* (collagen triple-helix repeat-containing 1), shows expression in the LH (ABA) as well as in the PVN and SON and it regulates lipid storage and cellular glycogen levels^27^. *Serpina3N*, the gene encoding the anti-protease alpha-1-antichymotrypsin (α_1_AC), is expressed most prominently in the Arc and weakly in the LH (ABA), while it has also been localized in the DMH and VMH and its expression in the hypothalamus was upregulated upon exposure to high-fat diet and leptin^28^.

#### Transcription factors

Besides the LH, the transcription factor *Asb4* is also expressed in the DMH and Arc (ABA) and has been identified in a GABAergic cluster of the LH^29^. This cluster also expressed *Sst* and *Col25a1*; nevertheless, there was no enrichment of these markers in LepR^LH^ neurons of our dataset, suggesting that LepR/Asb4 expressing cells form a separate population of neurons. *Dlx1*, a transcription factor involved in the production of Agrp and Ghrh neurons during development^30^, is expressed in the DMH, Arc, ZI and lightly in the LH (ABA).

#### GPCRs

Besides the Arc, the GPCR Y1 for Npy (*Npy1r*) is also expressed in the LH (ABA &^31^) and its signaling mediates NPY's effects on chow intake^32^. The LH receives input from Npy/Agrp cells in the Arc^33^, but also from Npy-expressing cells in the DMH^34^. It is also highly-probable that Npy1r^LH^cells receive input directly from Npy-expressing neurons resided in the LH, which are glucose-sensing^35^. Finally, another GPCR, *Gpr101*, is mainly expressed in the DMH, VMH, PVN as well as the LH (ABA), and its expression in the posterior hypothalamus is increased by fasting but decreased in the *ob/ob* mouse^36^.

A handful of genes with an association with energy balance were identified to be enriched in LepR^LH^ neurons and have proven expression in the LH and thus can be used as marker genes and be investigated further.

### LepR^LH^-enriched markers with widespread expression in the hypothalamus (ABA)

We continued exploring potential markers of LepR^LH^ neurons by looking at which genes have widespread expression in the hypothalamus based on ISH data (ABA) and have relevance with obesity and diabetes. The transcription factor *Isl1* has an important role in melanocortin neuron development in the hypothalamus^37^, and has been found to be associated with diabetes type 1 and 2^38,39^. The transporter *Cacna1e* has been associated with type 2 diabetes and obesity^40,41^. In the Arc, *Cacna1e* regulates leptin-induced excitation of POMC neurons with possible effects on hepatic insulin production and insulin resistance^42^ and it could potentially have a similar function in LepR^LH^ neurons. *Kdm6b*, a selective H3K27me3 demethylase, has widespread expression throughout the body. Its knock-out in RIP-Cre neurons in the Arc is associated with increased obesity^43^, suggesting that it may also regulate the function of LepR^LH^ neurons.

### Transcriptional response of TRAP-targeted cells to fasting

In order to determine the transcriptional response of LepR^LH^ neurons to food restriction, we fasted mice for 24 h before TRAP-seq and performed differential expression analysis between fed and fasted conditions. Weight gain as well as food and water intake were monitored before the fasting protocol, showing no differences between fed and fasted mice (Fig. S1a,b), while the 24-h food restriction caused significant weight loss (Fig. S1c). Differential expression (DE) analysis of LepR^LH^-enriched mRNA between fasted and fed mice revealed differences in gene expression levels induced by energy deficit (sup. Table 4).

It is known that 24-h fasting induces *Lepr* upregulation in the Arc and VMH^44^, while total hypothalamic expression of *Nts*, *Gal*, *Tac1* and *Cartpt* is not changed^45–47^. *Crh* is upregulated by fasting in the hypothalamus^47^, a change which is probably driven by the PVN, in which *Crh* is most prominently expressed. However, it is not known how these neuropeptides are regulated specifically in the LH or in LepR cells. Therefore, we examined whether food restriction would change the expression levels of the five neuropeptides as well as *LepR* in LepR^LH^-enriched samples from fasted and fed mice. There was no difference in *Lepr, Nts*, *Gal*, *Cartpt* or *Crh* expression levels, while *Tac1* was slightly upregulated by fasting (log2FoldChange = 0.84, *p* = 0.002).

**Figure 4:**
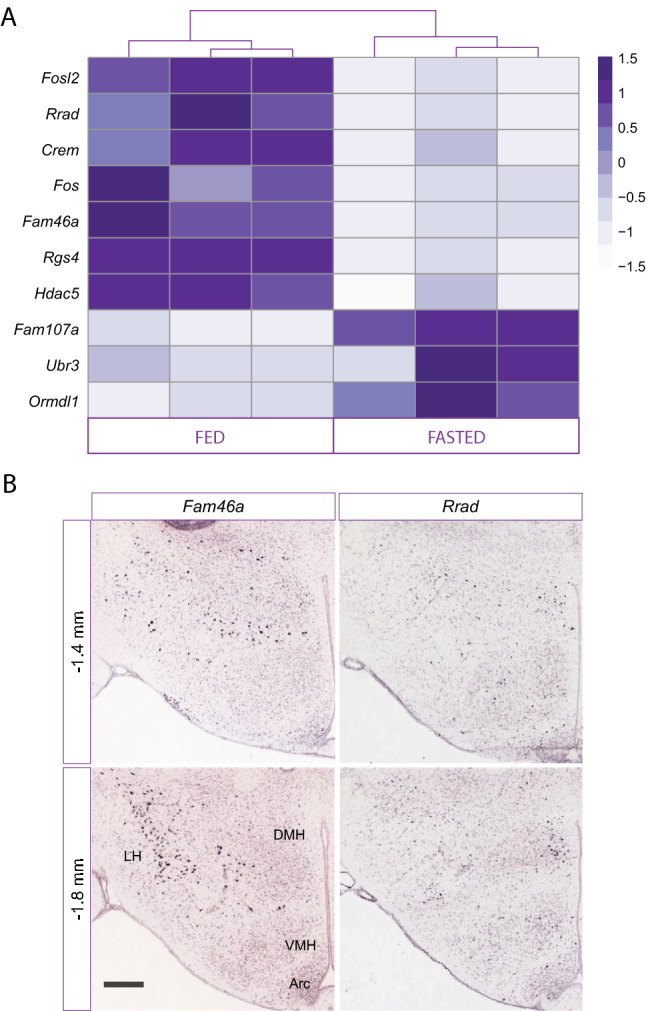
Genes differentially regulated by fasting (**A**) Heatmap of ten (10) genes that were differentially regulated by 24-h fasting (adjusted *p* < 0.05, n = 3 samples/condition). (**B**) Representative images of in situ hybridization (ISH) stainings for *Fam46a* and *Rrad* in the mouse hypothalamus (bregma points − 1.4 and − 1.8 mm) from the Allen Brain Atlas database.

When looking at the top regulated genes by fasting (with adjusted *p* < 0.05), there were 10 significantly DE genes with adjusted *p* < 0.05 out of which 3 were upregulated (*Fam107a*, *Ubr3a*, *Ormdl1*) and 7 were downregulated by fasting (*Fosl2*, *Rrad*, *Crem*, *Fos*, *Fam46a*, *Rgs4*, *Hdac5*) (Fig. 4a). Within the downregulated genes, we observed the immediate early gene *Fos*, confirming previous immunohistochemical data^15^, which show that food deprivation induces a decrease in the percentage of LepR^LH^ neurons that engage in cFos, from 12% in fed mice to 5% in fasted.

Fasting downregulated the expression of transcription factors *Fosl2*, *Crem*, *Hdac5*, all of which are associated with energy balance. *Hdac5* (Histone deacetylase 5) is a key epigenetic regulator of leptin's actions in the hypothalamus, by controling leptin signaling via STAT3 deacetylation, while it regulates POMC (and not Agrp) expression and protects against diet-induced obesity^48^. The transcription factor *Fosl2* promotes leptin gene expression in human and mouse adipocytes^49^. *Crem* belongs to the family of transcription factors CREB that promote insulin resistance in obesity in adipocytes^50^. It remains to be determined what role these transcription factors play in LepR^LH^ neurons.

The enzyme *Fam46a*, a translocator of leptin in adipocytes, was also downregulated by fasting. *Fam46a* is expressed prominently in the LH, PVN, SON and DMH (ABA) (Fig. 4b) and is associated with Body mass index (BMI)^51^. *Rgs4* (regulator of G-protein signaling-4) is a negative regulator of insulin release from pancreatic β-cells in vitro and in vivo (Ruiz de Azul et al., 2010, PNAS) and controls fatty acid and glucose homeostasis^52^, while its expression in the whole hypothalamus is downregulated by fasting^46^. Finally, *Rrad* (Ras Associated with Diabetes), which is expressed in the LH, DMH and VMH (ABA), is associated with type II diabetes by affecting insulin resistance and glucose intolerance^53^ and is overexpressed in the white adipose tissue of obesity-prone rats^54^. Although these proteins have been identified to affect body weight and to act in adipocytes and pancreatic β-cells, their role in LepR^LH^ neurons and link to regulation of energy balance remains to be determined.

Overall, food deprivation caused changes in the expression of many key players in energy balance and metabolic diseases. It would be interesting to characterize these genes as mediators of leptin's action in the LH and if and how they regulate neuropeptide expression as well as to unravel their role in energy homeostasis.

## Conclusion

Using viral RNA TRAP, we successfully profiled translated mRNAs of LepR^LH^ neurons. We identified several genes enriched in LepR^LH^ neurons with relevance to energy balance and metabolic diseases providing novel avenues for future studies. While expression of few of these genes (e.g. *Acvr1c*, *Npy1r, Itgb1, Cacna1e*) in the LH and in other hypothalamic nuclei has already been demonstrated, further characterization of their localization, function and their relation to leptin would provide insight on their metabolic function.

We also explored the transcriptional response of LepR^LH^ neurons to fasting. Neither *Lepr* nor four out of five established neuropeptides (*Nts*, *Gal*, *Cartpt*, *Crh*) were differentially regulated by food deprivation of 24 h, with *Tac1* being slightly increased. However, among the top DE genes, the transcription factors *Hdac5, Fosl2* and *Crem* were downregulated, indicating that food deprivation of 24 h induces changes in key transcriptional players. A longer food deprivation could potentially induce more robust transcriptional changes, although a 24 h fast is already a serious metabolic stress for a mouse. Nevertheless, exploring different fasting protocols that can be maintained for longer periods could add further knowledge to how LepR^LH^ neurons respond to energy deficit. Fasting also induced changes in expression levels of genes not previously reported to be affected in the whole hypothalamus with association to energy balance, such as the enzyme *Fam46a* as well as the GTP-binding protein *Rrad*, which are expressed prominently in the LH.

Overall, our dataset captures important information of rare cell types. We show that GABAergic LepR^LH^ neurons are diverse, reveal a set of genes that discriminate them from other LH cell types, and identify several obesity- and metabolism-related genes involved in their response to fasting. Our results can be the starting point of further characterization and studies about the involvement of this population, or its subpopulations, in diseases related to metabolism and energy balance.

## Methods

### Animals

Adult male ObRb-IRES-Cre mice (B6.129(Cg)-Leprtm2(cre)Rck/J) [LepR-Cre] and ObRb-IRES-Cre crossed with Rosa-CAG-LSL-tdTomato-WPRE::ΔNeo (008,320 & 007,914, Jackson laboratories, Bar Harbor, ME, US) [LepR-tdTomato] on C57Bl/6 J background were used. Mice were housed socially and kept under a 12:12 h light–dark cycle with lights off at 19:00. After surgery mice were single-housed and kept under a 12:12 light–dark cycle with lights off at 01:00. Mice were kept at room temperature (21 ± 2 °C) and 40–60% of humidity conditions. They were fed with standard chow (Special Diet Service, Essex, UK) and tap water ad libitum. For the 24 h of fasting protocol, chow was completely removed at 13:00—24 h (which always included the full 12 h dark period when normally most food intake occurs) before performing the TRAP protocol. All experiments were approved by the Animal Ethics Committee of Utrecht University and conducted in agreement with ARRIVE guidelines, Dutch laws (Wet op de Dierproeven, 1996; revised 2014) and European regulations (Guideline 86/609/EEC; Directive 2010/63/EU).

### AAV production

Plasmid pAAV-FLEX-EGFPL10a (kind gift from Alexander Nectow^17^) was packaged in AAV serotype 5 and rAAV1-FLEX-EGFPL10a was generated as described earlier^55^. Virus titer (number of genomic copies) was determined with RT-PCR with primers binding on the wPRE element.

### Surgeries

Two to three month-old ObRb-Cre (LepR-Cre) mice were anesthetized with ketamine (75 mg/kg, Narketan, Vetoquinol BV, Breda, The Netherlands) and medetomidine (1 mg/kg, Sedastart, AST Farma BV, Oudewater, The Netherlands). Mice were given eye cream (CAF, CEVA SanteAnimale BW, Naaldwijk, The Netherlands) and were placed on a stereotaxic apparatus (David Kopf Instruments, Tujunga, USA or Configuration Stereotaxic, 68U017, UNO, The Netherlands). A small incision was made along the midline of the skull and additional analgesia was applied by spraying Xylocaine (lidocaine 100 mg/ml, AstraZeneca BV, The Hague, The Netherlands) on the skull. 0.2 uL of rAAV1-FLEX-EGFPL10a (1–4 × 10^9 g.c. per uL) was injected bilaterally using a 34G stainless steel needle connected to a 10ul Hamilton syringe at a rate of 0.1 uL/min in the lateral hypothalamus (− 1.10 anteroposterior (AP), ± 1.90 mm mediolateral (ML) from Bregma, and − 5.30 mm dorsoventral (DV) from the skull, at an angle of 10°). After injection, the needle was maintained at its injection position for 15 min. After surgery, the animals were given carprofen for pain relief (5 mg/kg per day for 3 days, subcutaneous (s.c.)) and saline (0.4 ml/10gr, once, s.c.).

### TRAP protocol

The protocol previously published was followed^18^ and was performed in 7 batches of mice. 4 to 5 weeks after surgery, food was removed 1 h before starting the protocol. Mice were sacrificed between 13:00 and 13:30 by manual decapitation after isoflurane anesthesia. Brains were rapidly removed and kept in fresh dissection buffer (1 × HBSS, 2.5 mM HEPES pH 7.3, 35 mM Glucose, 4 mM NaHCO_3_, 100 ug/mL CHX). Hypothalami were microdissected, placed in homogenization buffer (10 mM HEPES pH 7.3, 150 mMKCl, 5 mM MgCl_2_, 100 ug/mL CHX, Complete-EDTA-free protease inhibitor (4,693,124, Roche), 1:100 RNasin RNase inhibitor(N2515, Promega), 1:100 Superasin RNase inhibitor(AM2696, Invitrogen, Carlsbad, CA, US) and 0.5 mM DTT) and homogenized in a tabletop homogenizer (E7000.25, Eberbach, St. Belleville, MI, US). The lysate was centrifuged for 10' at 2000 g at 4 degrees, NP-40 and freshly-dissolved DHPC were added to the supernatant at a final concentration of 1% and 30 mM and the supernatant was centrifuged for 15'at 20000 g at 4 degrees. 5% of the new supernatant was saved as input (hypothalamic) samples (IN) and the rest was incubated overnight (17–18 h) at 4 degrees with gentle end-over-end mixing in a tube rotator with Streptavidin MyOne T1 Dynabeads (65,601, Invitrogen) pre-coated with monoclonal anti-GFP antibodies(clones 19C8 and 19F7, Memorial Sloan-Kettering Monoclonal Antibody Facility, NY, US). Beads were collected on magnet and 5% of the supernatant was saved as unbound (UB) fraction. Beads were washed 4 times with high-salt buffer (10 mM HEPES KOH (pH 7.3), 350 mMKCl, 10 mM MgCl_2_, 1% NP-40, 100 ug/mL CHX, 1:100 RNasin RNase inhibitor and 0.5 mM DTT). RNA was washed off from beads (IP fraction) using lysis buffer with β-Mercaptoethanol from Absolutely RNA Nanoprep kit (400,753, Stratagene, San Diego, CA, US). All samples (IP, UB and IN) were purified with the Nanoprep kit following the manufacturer's instructions. RNA quality was assessed with Eukaryote total RNA Picochips on a 2100 Expert Bioanalyzer (Agilent, Santa Clara, CA, US). In total 13 samples (including IP, UB and IN fractions) were collected from fed and fasted mice with 4–6 hypothalami per sample. Two samples with RNA Integrity number (RIN) lower than 6.5 were excluded and 6 samples with very low RNA yield were merged by 2, resulting in 4 "fed" and 4 "fasted" samples used subsequently to generate cDNA libraries for RNA sequencing.

### CEL-Seq library preparation and Next-Gen Sequencing

A modified CEL-Seq protocol^56^ was used to generate CEL-Seq libraries. Briefly, 0,7–2,8 ng of IP and 10,8 ng of IN RNA were reverse-transcribed with Superscript II (18,064, Invitrogen) using barcoded polyT primers, followed by 2nd strand synthesis using DNA polymerase I (18,010,017, Thermo Fisher Scientific), ThemoFisher Scientific). The cDNA was in vitro transcribed using the T7 Megascript kit (AM1334, Invitrogen). RNA was fragmented in 75'' at 94 degrees in fragmentation buffer (200 mM Tris–acetate, pH 8.1, 500 mM KOAc, 150 mM MgOAc) and stopped with Fragmentation stop buffer (0.5 M EDTA). RNA quality and amount was assessed with Eukaryote total RNA Picochips on a 2100 Expert Bioanalyzer. 1.7–16.8 ng of RNA underwent reverse transcription followed by PCR amplification of 10–15 cycles with uniquely indexed RNA PCR primers (Illumina rpi primers) assigned to each sample. cDNA quality was assessed with High Sensitivity DNA Assay on a Bioanalyzer and concentrations were assessed with Qubit (Thermofisher, Waltham, MA, US). Libraries were sequenced on an Illumina NextSeq500 using 1 × 75 bp high output end sequencing modified for paired-end sequencing (26-6-60 read1-index-read2).

### RNA sequencing analysis

RNA-seq reads were aligned to annotated exons using the mm10mouse reference genome and to the L10:GFP sequence using bwa mapping. Reads that did not align or aligned to multiple locations were discarded. Two samples (one from fed and one from fasted groups) were removed: in the former sample the IP fraction had very low number of reads and in the latter sample the IP fraction had extremely high amount of reads for *LepR*. Mitochondrial genes and gene Rn45s were excluded. DESeq2^57^ was used to normalize the reads according to the dispersion profile of the whole dataset and to perform differential expression analysis between LepR^LH^ and hypo fed samples as well as between fed and fasted in IP samples. We calculated fold-enrichment as normalized reads in LepR^LH^ divided by normalized reads in hypo samples. For the LepR^LH^ and hypo comparison, enriched genes in either the IP or the IN fraction were filtered for an adjusted p value cutoff of < 0.01 and |log2| fold change > 1.5. For annotating the enriched genes in IP, we used online databases for secreted proteins (Uniprot), transcription factors (Riken TFdb) and GPCRs, catalytic receptors, enzymes, ion channels and transporters (IUPHAR). For the fed and fasted comparison, differentially expressed genes were filtered for an adjusted *p* value cutoff of < 0.05.

### Histology

Two to three weeks after surgery animals were sacrificed with sodium pentobarbital overdose (200 mg/mL, Euthanimal, Alfasan BV, The Netherlands). Animals were perfused with ice-cold 1 × phospate buffered saline (PBS) pH 7.3, followed by ice-cold 4% paraformaldehyde (PFA) in 1 × PBS pH 7.3. Brains were removed and incubated for 6 h or overnight in 4% PFA for in situ hybridization and immunofluorescence respectively, then transferred consecutively to 20% for 1 day and 30% sucrose solution (in 1 × PBS) for 2 days. Brains were snap-frozen by isopentane immersion and stored at − 80 °C. Coronal sections of the hypothalamus were sliced in a cryostat (Leica, Wetzlar, Germany). For in situ hybridization 20 um sections were mounted on Superfrost slides and stored at − 80 degrees, whereas for immunofluorescence 40 um free-floating sections were collectedin PBS 1 × with Sodium Azide 0.01% and stored at 4 degrees.

### Immunofluorescence for GFP

Free-floating sections were incubated with blocking solution (10% normal goat serum (NGS), 1% Triton X-100 in 1 × PBS) for 1 h at room temperature (RT), followed by overnight incubation at 4 degrees with chicken anti-GFP (ab13970, Abcam, Cambridge, UK) 1:1000 in carrier solution (3% NGS, 0,25% Triton X-100 in 1 × PBS). Sections were then incubated with the goat anti-chicken 488 (ab150169, Abcam, Cambridge, UK) 1:500 in carrier solution for 1 h at RT and in DAPI (1:1000 in PBS 1x) for 15–30 min at RT. Between all steps sections were washed 3 times for 5–10 min in PBS 1x. Sections were then mounted on microscope glasses, let to dry and covered with Fluorsave (Calbiochem, San Diego, CA, US).

### Cloning and DIG probe labeling

PCR with forward and reverse primers (see Table 1) was performed on mouse hypothalamic cDNA (isolated with miRNeasy Mini Kit, Qiagen, Hilden, Germany). PCR products were ligated into pGEMT.easy (Promega, Madison, WI, US) and sequenced with Sanger sequencing. PCR with SP6 and T7 primers was performed. cDNA probes were incubated for 2 h at 37 degrees with Dig RNA labeling mix (11,277,073,910, Merck, Germany) and SP6 or T7 RNA polymerase (1,487,671 and 881,775, Roche, Basel, Switzerland).

### Fluorescent in situ hybridization and immunofluorescence for tdTomato

Slides with 20 um hypothalamic sections were thawed at room temperature (RT) for 1 h. Sections were incubated with 1,32% triethanolamine and 0,18% HCl for 10' at RT and with hybridization mix (50% deionized formamide, 5 × SSC buffer, 5 × Denharts, 250 μg/ml tRNA baker's yeast and 500 μg/ml Sonificated Salmon Sperm DNA) for 2 h at RT. Sections were incubated with RNA probes (400 ng/mL hybridization mix) overnight at 68 degrees. Endogenous tdTomato signal was not detectable after this stage (Data not shown). Slides were transferred to 2 × SSC at 68 degrees and then immediately to 0.2 × SSC at 68 degrees for 2 h. After that, sections were treated with 0.3% hydrogen peroxide in 1 × TBS for 30' at RT, blocked with TNB blocking buffer (from TSA Plus Cyanine 3 System, NEL744001KT, PerkinElmer, Waltham, MA, US) for 1 h at RT and incubated with anti-DIG-POD (1:500, 11,207,733,910, Roche) and rabbit anti-RFP (1:1000, 600-401-379, Rockland, Limerick, PA, US) in TNB blocking buffer overnight at 4 degrees. Sections were then incubated with Cyanine 3 Tyramide amplification reagent (1:50 in 1X amplification diluent) from TSA kit for 10–15' at RT followed by incubation with goat anti-rabbit 568 (1:500, ab175471, Abcam, Cambridge, UK) in TNB blocking buffer and DAPI (1:1000 in 1 × PBS). Between steps sections were washed 4 × 5' with 1xTNT buffer (0,1 M Tris–HCl, pH7.5, 0.15 M NaCl, 0.05% Tween20). Sections were let to dry and covered with Fluorsave reagent (Calbiochem, San Diego, CA, US).

### Imaging and image analysis

For each batch of surgeries, we confirmed expression of GFP in the LH. However, it was not possible to detect GFP expression under fluorescent light during the dissection, probably due to the low number of targeted cells and the autofluorescence of the tissue. Therefore, to assess expression of GFP in the batches of mice that were used for TRAP, the brains of 1 or 2 mice per batch were fixed 2 weeks after surgery and hypothalamic sections were processed in order to immunohistochemically detect GFP (see below). At the microscope we confirmed that most of GFP cells were located in the LH. For quantifications of GFP expression, 10 × magnification pictures were taken with an epi-fluorescent microscope (Zeiss Scope A1, ZEISS, Germany). Five sections with an interval of 0,2–0,3 mm ranging from − 1.1 to − 2.4 mm caudal to bregma (as indicated in^58^), were selected. GFP cells in the LH, ZI and PeF were manually counted using the Cell Counter plugin in ImageJ. For quantifications of co-localized cells regarding FISH for neuropeptides and tdTomato IHC, the same settings were used, except that 20 × magnification of the LH, ZI, PeF, Tu were taken. We quantified the total number of tdTomato cells located in the LH/ZI/PeF/Tu and the number of tdTomato cells co-expressing the corresponding neuropeptide, in order to calculate the percentage of LepR^LH^ cells expressing each neuropeptide.

### Statistical analyses

Regarding body weight and food intake data, statistical analyses were performed with GraphPad Prism 7.0 (Graphpad Software, San Diego, CA, US). Data was checked for normality before doing statistical tests.

## Supplementary Information

Supplementary Information 1.

Supplementary Information 2.

Supplementary Information 3.

Supplementary Information 4.

Supplementary Information 5.

## Data Availability

All data generated and analysed during this study are included in this article and its supplementary information files. Additional information related to this study can be obtained from the corresponding author on reasonable request.
