# Supplementary material for: Molecular profile and response to energy deficit of leptin-receptor neurons in the lateral hypothalamus
Source: Sci Rep. 2022 Aug 4;12:13374. doi: 10.1038/s41598-022-16492-w (PMC9352899; doi:10.1038/s41598-022-16492-w)
Supplement: Supplementary file 1 — Supplementary Information 1. [file 41598_2022_16492_MOESM1_ESM.pdf]

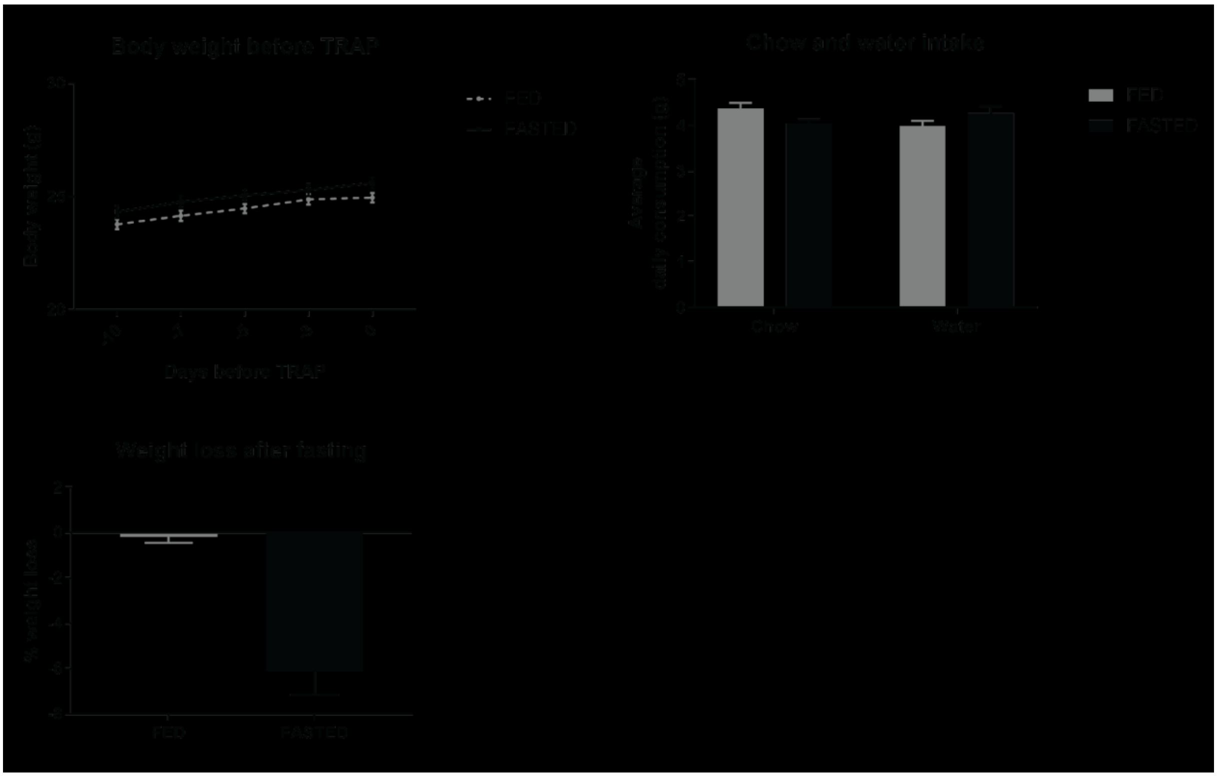

Figure S1 Food intake and body weight before TRAP. **A** Body weight of mice measured every 2-3 days, starting 10 days before TRAP up to 1 day before TRAP (two-way ANOVA: no effect). **B** Average daily consumption of chow and water starting 10 days before TRAP up to 1 day before TRAP (individual unpaired t-tests for chow: no effect, water: no effect). **C** Percentage of body weight change between 1 day before and TRAP day in fed and fasted animals (two-way ANOVA; interaction effect (day x condition): F (1, 59) = 29,78, p < .0001; post-hoc Sidak's test: p = 0,9747 for fed (n=28), p < .0001 for fasted mice (n=33)). Data presented as mean±SEM
